# Outcomes of brentuximab vedotin in cutaneous T-cell lymphoma: A Middle Eastern real-world study

**DOI:** 10.1016/j.jdin.2026.07.011

**Published:** 2026-08-14

**Authors:** Ahmad Alsarraf, Hashem Boalbanat, Jihan Rajy, Karen Pinto, Dina Elkady, Rasha Abdel Tawab Hamed

**Affiliations:** a Kuwait Institute of Medical Specialization, Kuwait City, Kuwait; b As’ad Al-Hamad Dermatology Center, Kuwait City, Kuwait; c Kuwait Cancer Control Center, Kuwait City, Kuwait

**Keywords:** advanced-stage cutaneous T-cell lymphoma, antibody-drug conjugate, brentuximab vedotin, CD30, cutaneous T-cell lymphoma, Middle East, mycosis fungoides, overall survival, primary cutaneous anaplastic large-cell lymphoma, progression-free survival, real-world evidence, real-world study, retrospective cohort study, Sézary syndrome, time to next treatment, treatment outcomes

*To the Editor:* Brentuximab vedotin (BV) is an established therapy for relapsed or refractory CD30-positive cutaneous T-cell lymphoma (CTCL), supported by the ALCANZA randomized and real-world data from Western and Eastern populations.^1^, ^2^, ^3^, ^4^ However, real-world data from Middle Eastern populations are lacking. We evaluated the real-world effectiveness and safety of BV in a Middle Eastern cohort of patients with CTCL.

We conducted a retrospective study of adult patients with histologically confirmed CTCL treated with BV at the Kuwait Cancer Center between 2018 and 2025. Clinical characteristics, CTCL subtype, staging, and treatment response were reviewed and classified according to established consensus criteria.^5^ Statistical analyses were conducted using R (version 4.3.2; R Foundation for Statistical Computing).

Nineteen patients were included (median age, 52 years; range, 16-78 years), with a predominance of advanced-stage disease (84%) and mycosis fungoides subtype (89%). Median CD30 expression was 20%. While 21% patients were treatment-naïve, most patients (63%) had received ≥3 prior lines of therapy (Supplementary Table I, available via Mendeley at https://data.mendeley.com/datasets/7fbh39f94j/1). Baseline characteristics are summarized in Table I.

**Table I:** Baseline demographic and clinical characteristics of patients treated with brentuximab vedotin

Variable	Values (*n* = 19)
Age (y)	
Median (range)	52 (16-78)
Sex, *n* (%)	
Female	7 (37%)
Male	12 (63%)
Clinical stage, *n* (%)	
Early stage	3 (16%)
IB	1 (5%)
IIA	2 (11%)
Advanced stage	16 (84%)
IIB	10 (53%)
IIIA	1 (5%)
IIIB	1 (5%)
IVA1	2 (11%)
IVA2	2 (11%)
Disease subtype, *n* (%)	
Mycosis fungoides	17 (89%)
Sézary syndrome	2 (11%)
No. of treatment lines received prior to BV, *n* (%)	
0	4 (21%)
1	1 (5%)
2	2 (11%)
≥3	12 (63%)
CD30%	
Median (range)	20% (5%-80%)
<10%	2 (11%)
10%-30%	10 (53%)
≥30%	6 (32%)
NA	1 (5%)

*BV,* Brentuximab vedotin; *NA*, not available.

Our patients received a median of 13 (range, 5-16) BV cycles. The objective response rate was 68%, including 53% complete and 16% partial responses, while progressive disease occurred in 32% of patients during treatment, as demonstrated in Table II. Median progression-free survival was 17 months (95% CI, 6-not reached), and median time to next treatment was 21 months (95% CI, 9-not reached). Patients with stage III or higher disease appeared to have shorter median time to next treatment than those with earlier stage disease (8 vs 22 months; log-rank *P* = .03). Median overall survival was not reached; estimated 3-year overall survival was 71.8%. Peripheral neuropathy was the most common adverse event, occurring in 47% of patients (grades 2-3), and was managed with dose modification. Two patients experienced severe treatment-related pulmonary complications requiring intensive care (one with COVID-19 pneumonia and the other with interstitial pneumonitis).

**Table II:** Objective response rate by patient subgroup

Variable	Total	Objective response rate, *n* (%)	CR	PR	PD
Overall	19	13 (68%)	10 (53%)	3 (16%)	6 (32%)
Stage					
IA-IIA	3	3 (100%)	1 (33%)	2 (67%)	0 (0%)
IIB	10	8 (80%)	2 (20%)	6 (60%)	2 (20%)
IIIA-IIIB	2	0 (0%)	0 (0%)	0 (0%)	2 (100%)
IV	4	2 (50%)	0 (0%)	2 (50%)	2 (50%)
No. of prior treatment lines					
<3	7	4 (57%)	0 (0%)	4 (57%)	3 (43%)
≥3	12	9 (75%)	3 (25%)	6 (50%)	3 (25%)
CD30					
<10%	2	2 (100%)	1 (50%)	1 (50%)	0 (0%)
10-30	10	6 (60%)	2 (20%)	4 (40%)	4 (40%)
≥30	6	5 (83%)	0 (0%)	5 (83%)	1 (17%)
NA	1	0 (0%)	0 (0%)	0 (0%)	1 (100%)

*CR*, Complete response; *NA*, not available; *PD*, progressive disease; *PR*, partial response.

Compared with the ALCANZA trial population, our cohort was younger (median age, 52 vs 62 years), had a greater proportion of advanced-stage disease (84% vs 67%), and included patients with CD30 expression levels below 10% and a subset of treatment-naïve patients, reflecting routine practice. Despite these differences, our outcomes were comparable to those reported in the ALCANZA trial.^1^ Real-world studies predominantly included advanced-stage disease, did not include treatment-naïve patients, and variably included CD30 expression levels below 10%. Overall, our results were within the range reported by these international studies, which demonstrated objective response rates of approximately 64% to 92%, median progression-free survival of approximately 7 to 16.7 months, and peripheral neuropathy rates between 25% and 57%.^2^, ^3^, ^4^

This study is limited by its retrospective and single-center design. Given the modest sample size, subgroup findings should be considered descriptive only and interpreted with caution. In addition, follow-up duration remains insufficient to fully characterize long-term survival outcomes.

In this Middle Eastern real-world cohort, BV demonstrated clinical activity and an acceptable safety profile in patients with CTCL. Notably, our cohort included patients with a broader range of CD30 expression and a subset of treatment-naïve patients, reflecting real-world practice patterns. These findings add regional real-world evidence that is broadly consistent with previously reported BV outcomes.

### Declaration of generative AI and AI-assisted technologies in the writing process

During the preparation of this work the authors used ChatGPT (OpenAI) in order to improve grammar, language clarity, and sentence flow. After using this tool, the authors reviewed and edited the content as needed and take full responsibility for the content of the publication.

## Conflicts of interest

Dr Tawab Hamed reports receiving speaker honoraria from F. Hoffmann-La Roche, Kyowa Kirin, Sobi, MSD, and Takeda. Drs Alsarraf, Boalbanat, Rajy, Pinto, and Elkady have no conflicts of interest to declare.
